# Clinical and Genetic Spectrum of ANO3-Related Dystonia with Treatment Responses in a Chinese Cohort

**DOI:** 10.3390/genes17060703

**Published:** 2026-06-17

**Authors:** Jie-Hong Huang, Yang Li, Zhi-Meng Wan, Li-Xi Li, Ling-Jing Jin, Xin-Hua Wan

**Affiliations:** 1 Department of Neurology and Neurological Rehabilitation, Shanghai Disabled Persons’ Federation Key Laboratory of Intelligent Rehabilitation Assistive Devices and Technologies, Yangzhi Rehabilitation Hospital (Shanghai Sunshine Rehabilitation Center), School of Medicine, Tongji University, Shanghai 201619, China; 2 Neurotoxin Research Center of Key Laboratory of Spine and Spinal Cord Injury Repair and Regeneration of Ministry of Education, Department of Neurology, Tongji Hospital, School of Medicine, Tongji University, Shanghai 200065, China; 3 Department of Neurology, Peking Union Medical College Hospital, Chinese Academy of Medical Sciences and Peking Union Medical College, Beijing 100730, China; 4 Collaborative Innovation Center for Brain Science, Tongji University, Shanghai 200092, China

**Keywords:** *ANO3*, dystonia, whole-exome sequencing

## Abstract

**Background:** Variants in anoctamin 3 (*ANO3*) are linked to autosomal dominant dystonia, commonly known as DYT-*ANO3* (OMIM: #615034). While craniocervical dystonia constitutes the most frequently observed phenotype, its clinical manifestations display significant heterogeneity. Nevertheless, the data concerning the genetic characteristics, clinical features, and therapeutic outcomes of ANO3-related dystonia within Asian populations remain scarce. **Methods:** Whole-exome sequencing was conducted on 661 Chinese patients, comprising 356 individuals with dystonia and 305 individuals with non-dystonic movement disorders who served as an internal disease–control cohort. Candidate *ANO3* variants were evaluated based on population frequency, predicted deleteriousness, ACMG criteria, and aggregate frequency analyses. A retrospective review was performed of clinical features and treatment responses. **Results:** Fifteen rare *ANO3* missense variants were identified in 16 patients with dystonia and one non-dystonic individual, including one pathogenic variant, six likely pathogenic variants, and nine previously unreported variants. The rare *ANO3* variants were significantly enriched in the dystonia cohort compared with the controls. ANO3-related dystonia exhibited broad clinical heterogeneity, with frequent cervical involvement (62.5%) and tremulous features (75%), and occasionally extended beyond classical isolated dystonia. Oral medication and botulinum toxin showed variable benefit, whereas deep brain stimulation was associated with marked improvement in selected medically refractory patients. **Conclusions:** This study broadens the genetic and clinical spectrum of ANO3-related movement disorders in a Chinese cohort. The findings support substantial clinical heterogeneity in DYT-*ANO3* and suggest that deep brain stimulation, especially STN-DBS, may be considered in selected refractory cases, although further studies are needed.

## 1. Introduction

Dystonia constitutes a collection of movement disorders marked by persistent or intermittent muscle contractions that produce abnormal movements or postures, frequently manifesting as patterned or twisting motions [1]. However, the phenomenology of dystonia is highly heterogeneous, rendering classification based on clinical features challenging [2]. Consequently, screening for pathogenic genes associated with DYT remains a crucial endeavor in the quest to identify etiological factors [3].

In 2013, Charlesworth et al. identified mutations in the *ANO3* gene linked to familial cervical dystonia, now classified as DYT-24 (DYT-*ANO3*; OMIM: #615034) [4]. Subsequent studies have shown that ANO3-related disease most commonly presents with focal or segmental dystonia involving the craniocervical region, but its clinical spectrum has gradually expanded to include generalized dystonia, tremulous dystonia, myoclonus, parkinsonian features, and other hyperkinetic manifestations [5,6,7,8,9].

Numerous clinical and genetic characterizations of DYT-*ANO3* in patients of European descent exist, with reports involving Asian populations remaining limited [5,9,10,11,12,13,14,15,16,17]. The evidence on therapeutic responses, long-term prognosis, and optimal strategies is scarce. To date, no comprehensive study has systematically characterized the genetic and phenotypic features of DYT-*ANO3* within a Chinese population.

The treatment data for ANO3-related dystonia are still limited [8,9]. The reported responses to oral medications are variable, and potential benefits from dopaminergic agents, anticholinergics, or antiepileptic drugs have mainly been described in individual patients or small series [8]. Deep brain stimulation, particularly globus pallidus internus DBS, has also been reported to be beneficial in selected cases [13,18,19,20] Nevertheless, systematic evidence on treatment response remains sparse, especially for DBS.

In this study, we performed genetic screening of a multicenter Chinese cohort comprising 356 patients with dystonia and 305 patients with non-dystonic movement disorders. We aimed to characterize the clinical and genetic spectrum of ANO3-related movement disorders; delineate their clinical heterogeneity; and summarize the treatment responses to oral medication, botulinum toxin, and deep brain stimulation.

## 2. Subjects and Methods

### 2.1. Participants

A total of 356 patients with dystonia were consecutively recruited from the outpatient and inpatient neurology departments at Peking Union Medical College Hospital (*n* = 273) and Tongji Hospital, Tongji University School of Medicine (*n* = 83) (DYT cohort) between October 2019 and November 2025.

Furthermore, 305 patients presenting with non-dystonic movement disorders were enrolled during the same timeframe, forming an internal disease–control cohort (non-DYT cohort) for an aggregate frequency analysis. All individuals in the non-DYT cohort were assessed by experienced neurologists specializing in movement disorders and showed no clinical signs of dystonia. The diagnostic composition of this cohort is detailed in Table S1.

Each patient underwent a detailed medical history, neurological examination, and assessment using the dystonia classification data collection form by at least two experienced neurologists. DYT diagnoses were made following the International Parkinson and Movement Disorder Society Dystonia Task Force guidelines to ensure diagnostic accuracy [1,2]. The study was approved by the Ethics Committee of Peking Union Medical College Hospital and Tongji Hospital, Tongji University School of Medicine, and it was conducted in accordance with the Declaration of Helsinki, with written informed consent obtained from all participants.

### 2.2. Whole-Exome Sequencing and Variant Evaluation

A total of 661 unrelated patients underwent whole-exome sequencing (WES) to identify rare variants in *ANO3*. Genomic DNA was isolated from peripheral blood samples anticoagulated with EDTA. Exome capture and paired-end high-throughput sequencing were conducted on an Illumina sequencing platform. The raw reads underwent quality control, and the clean reads were aligned to the human reference genome GRCh37/hg19 using Burrows Wheeler Aligner (BWA version 0.7.12-r1039). Duplicates were marked, and variant calling was performed using a Genome Analysis Toolkit (GATK version 3.7). Variants were subsequently annotated with ANNOVAR (v2016-02-01). The detailed methodologies for the WES are provided in the Supplementary Material File S1.

**Figure 1:**
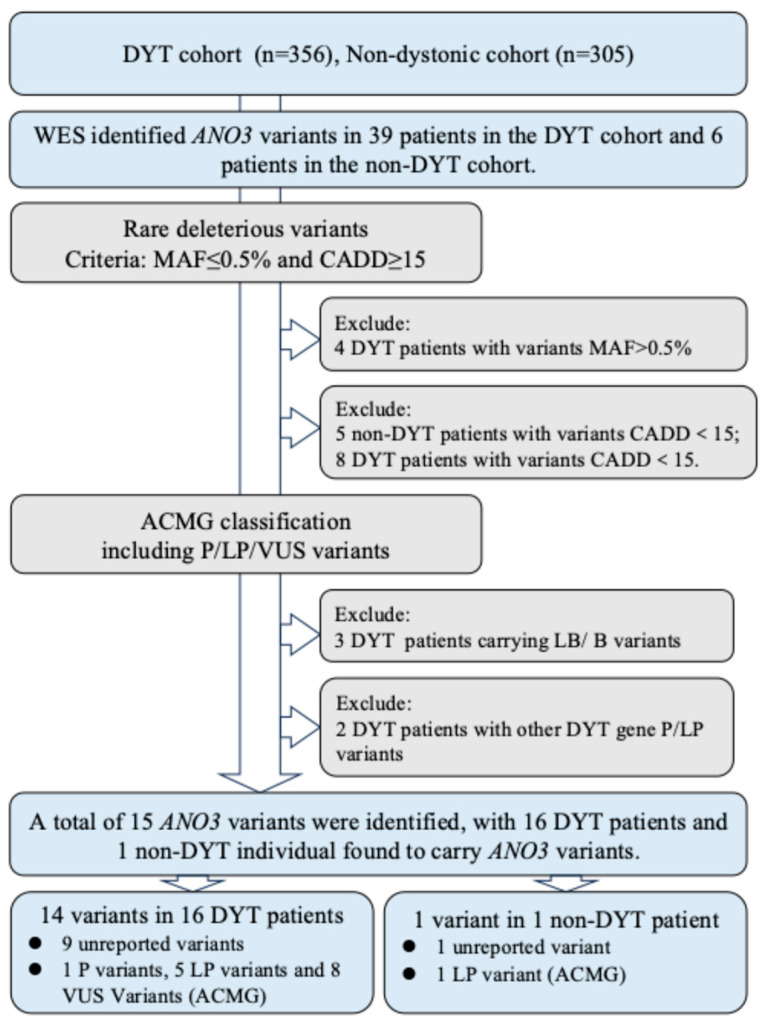
Research workflow of the present study. ACMG, American College of Medical Genetics and Genomics; DYT, dystonia; MAF, minor allele frequency; CADD, combined annotation-dependent depletion; P/LP, pathogenic or likely pathogenic; LB/B, likely benign or benign; VUS, variant of uncertain significance; WES, whole-exome sequencing.

Since *ANO3* is a dominantly inherited gene, variants with an allele frequency less than 0.5% (gnomAD-all, gnomAD-EAS, and HUABIAO databases) were retained, including heterozygous variants [21,22]. The analysis focused on predicted pathogenic nonsense, frameshift, non-frameshift, missense, and potential splice-site variants within ±2 bp of exon–intron boundaries. Variant pathogenicity was initially annotated with InterVar and then reviewed per the ACMG/AMP guidelines. The variants classified as pathogenic, likely pathogenic, or of uncertain significance (VUSs) were retained for further analysis. The final ACMG classifications and evidence codes are summarized in Table 1 [23,24]. For the variant analysis, individuals carrying pathogenic or likely pathogenic (P/LP) variants in other DYT-related genes were excluded. The candidate variants were validated by Sanger sequencing and cross-referenced with MDSGene (https://mdsgene.org/, accessed on 10 December 2025), PubMed, and ClinVar to confirm prior clinical relevance. The study workflow is outlined in Figure 1.

Meanwhile, the damaging effect of candidate missense variants was predicted utilizing the online in silico tools Varcards2 (https://genemed.tech/varcards2/, accessed on 15 December 2025) [25]. The predictions from SIFT, PolyPhen-2, MutationTaster, MutationAssessor, MetaSVM, CADD, GERP++, phastCons, SiPhy, and additional available tools were evaluated via the VarCards2 interface. Missense variants with a CADD score below 15 were deemed to have relatively low predicted deleteriousness and were consequently excluded from the subsequent analysis. To investigate the potential molecular mechanisms, LOGOFunc (https://itanlab.shinyapps.io/goflof/, accessed on 4 June 2026) was used to predict the most likely functional consequence of each rare *ANO3* missense variant, which was classified as neutral, gain-of-function (GOF), or loss-of-function (LOF). The category with the highest probability score was designated as the predicted functional mechanism.

For the aggregate frequency analysis, rare deleterious *ANO3* variants were consolidated at the gene level and compared between the dystonia cohort and control datasets. Qualifying variants encompassed rare protein-altering or canonical splice-site variants with a minor allele frequency (MAF) of less than 0.5%, which were classified as pathogenic, likely pathogenic, or variants of uncertain significance according to the ACMG criteria. Missense variants with CADD scores below 15, synonymous variants, likely benign/benign variants, and low-quality variants were excluded from the analysis. The allele counts were compared utilizing Fisher’s exact test, and odds ratios with 95% confidence intervals were calculated. The internal non-DYT cohort was regarded as the most comparable control group, whereas the gnomAD-all, gnomAD-EAS, and HUABIAO databases (https://www.biosino.org/, accessed on 4 April 2026) served as external population reference datasets [26].

### 2.3. Clinical Data Collection and Treatment Outcome

Clinical data were collected through face-to-face interviews by trained neurologists. Patient demographics, age at symptom onset, disease duration, dystonia subtype, affected body regions, and family history were recorded. Standardized physical examinations and functional assessments—including drinking, pouring water, arm extension, fanning, finger-to-nose testing, writing, and spiral drawing—were performed, with upper limb evaluation conducted independently. Data were entered into an electronic record system and were used to review medication history, botulinum toxin treatments, and DBS plans. The baseline assessment used the Burke–Fahn–Marsden Dystonia Rating Scale (BFMDRS) to evaluate dystonia severity [27]. The BFMDRS evaluations were conducted during clinical visits by movement disorder specialists who had received standardized training. Due to the retrospective design of this study, the BFMDRS assessments were not carried out under blinded conditions; however, the evaluating clinicians were unaware of the specific research objectives of the present study at the time of clinical assessment. The patients were re-evaluated after treatment during follow-up visits, or via video if in-person visits were not possible. Improvement (%) was calculated as [(baseline score − post-treatment score)/baseline score] × 100%.

### 2.4. Statistical Analysis

The data were analyzed and visualized using PyCharm software (v 2024.3.6 Professional Edition based on Python 3.12) and GraphPad Prism (version 10.1.1). Student’s *t*-tests were employed to compare two independent samples, whereas Mann–Whitney U tests were utilized for nonparametric data. A one-way ANOVA or Kruskal–Wallis test was used to compare the means of multiple groups. Statistically significant results were defined as those with *p* values less than 0.05.

## 3. Results

### 3.1. Identification of ANO3 Variants

*ANO3* variant identification was performed according to the study workflow shown in Figure 1. Among 356 unrelated patients with dystonia, 39 individuals carrying 29 distinct *ANO3* variants were identified, including 21 missense and six splice-site variants. In the non-DYT cohort of 305 unrelated patients with other movement disorders, four distinct *ANO3* variants were detected in six individuals, comprising one missense and three splice-site variants.

During variant evaluation, two patients carrying *ANO3* variants were excluded from the ANO3-focused analysis because both also harbored pathogenic or likely pathogenic variants in THAP1, a known dystonia-associated gene. One patient carried *ANO3* c.1747A > G (NM_031418; VUS) and THAP1 c.71 + 1G > A (NM_018105; likely pathogenic), while the other carried *ANO3* c.771G > C (NM_031418; VUS) and *THAP1* c.63_66del (NM_018105; pathogenic).

In addition, four individuals were excluded because they carried variants with a minor allele frequency exceeding 0.5%, and 13 individuals were excluded because they carried variants with a CADD score below 15. Furthermore, three DYT patients carrying variants classified as benign or likely benign were excluded in accordance with the ACMG criteria. The specific exclusion details and WES secondary findings are delineated in Supplementary Material File S2.

The pedigrees of 17 cases with *ANO3* variants, along with their corresponding phenotypes and DNA sequencing chromatograms of the *ANO3* variants, are provided in Figures S1 and S2. A total of eight trios were subjected to WES, resulting in the identification of one de novo mutation and one instance of paternal inheritance. In the remaining eight families, the carriers demonstrated asymptomatic presentation, regardless of paternal or maternal lineage.

### 3.2. Genetic Features of ANO3 Variants

**Figure 2:**
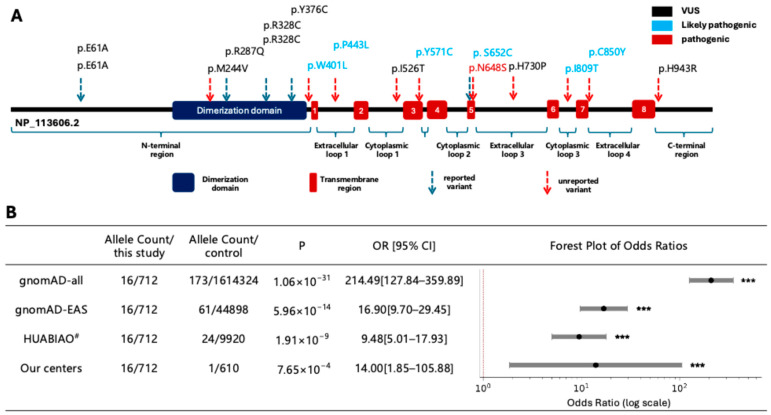
Genetic features and aggregate frequency analysis of *ANO3* variants. (**A**) The positions of 15 rare variants in the *ANO3* gene identified among 17 patients are depicted. The red text denotes pathogenic variants; the blue text indicates variants that are likely pathogenic; and the black text represents variants of uncertain significance. The red arrows indicate novel variant of *ANO3* identified in our cohort. (**B**) The aggregated frequency analysis of *ANO3* variants in the dystonia cohorts in comparison to control groups. *p* values and odds ratios are estimated using a two-sided Fisher’s exact test. CI, confidence interval; gnomAD-all, Genome Aggregation Database All population, gnomAD-EAS, Genome Aggregation Database East Asian; OR, odds ratio; VUS, variant of uncertain significance; ^#^ HUABIAO, HUABIAO project: public database of whole-exomes of the Han Chinese; *** *p* < 0.001.

After filtering, a total of 15 variants were identified from 661 patients in our cohort. A total of 14 rare missense *ANO3* variants were identified in 16 unrelated patients with dystonia, including nine variants that have not been previously reported in association with dystonia (Figure 2A). Five variants were classified as likely pathogenic, all of which were previously unreported (p.W401L, p.Y571C, p.S652C, p.I809T, and p.C850Y). One de novo variant was classified as pathogenic (p.N648S).

Three variants (p.H730P, p.I809T and p.C850Y) were absent in the gnomAD database, whereas the remaining variants demonstrated very low allele frequencies (MAF < 5 × 10^−3^). The i silico pathogenicity and functional mechanism predictions of the rare *ANO3* missense variants are summarized in Table S2. All the included variants had CADD scores above 15, and eight of the 14 dystonia-associated variants were predicted to be pathogenic by at least five computational algorithms.

LOGOFunc analysis was conducted to evaluate the potential functional implications of the identified *ANO3* missense variants. Three variants, p.R287Q, p.N648S, and p.S652C, were predicted to align more closely with a gain-of-function (GOF) effect, while p.Y376C, p.I809T, and p.P443L were suggested to correspond to a loss-of-function (LOF) effect. The remaining variants were anticipated to be neutral. These findings imply possible mechanistic heterogeneity among the *ANO3* missense variants. Nonetheless, these in silico predictions should be interpreted with caution and do not constitute functional validation.

In the non-DYT cohort, a previously unreported *ANO3* missense variant (p.P443L) was identified and was absent from both gnomAD and HUABIAO. This variant was classified as likely pathogenic in accordance with the ACMG criteria (PM1, PM2, PP2, PP3), supported by pathogenic predictions from more than ten in silico tools (see Table S2).

The variants were distributed across various functional domains of the *ANO3* gene: two within the N-terminal region (p.E61A, p.W401L); four within the dimerization domain (p.M244V, p.R287Q, p.R328C, p.Y376C); two LP/P variants within transmembrane domain 5 (p.N648S, p.S652C); and one variant each in extracellular loop 1 (p.P443L), cytoplasmic loop 1 (p.I526T), transmembrane domain 3 (p.Y571C), extracellular loop 3 (p.H730P), cytoplasmic loop 3 (p.I809T), extracellular loop 4 (p.C850Y), and the C-terminal region (p.H943R) (see Figure 2A).

A burden analysis demonstrated that the overall frequency of *ANO3* variants in patients with dystonia was significantly higher when compared to population controls, including gnomAD-all (*p* = 1.06 × 10^−31^; OR = 214.49; 95% CI: 127.84–359.89), East Asian populations (*p* = 5.96 × 10^−14^; OR = 16.90; 95% CI: 9.70–29.45), and Chinese populations (*p* = 1.91 × 10^−9^; OR = 9.48; 95% CI: 5.01–17.93) (See Table S3, Figure 2B). Likewise, the prevalence of heterozygous *ANO3* variants was markedly increased in the DYT cohort in comparison to the non-DYT cohort (*p* = 7.65 × 10^−4^; OR = 14.00; 95% CI: 1.85–105.88) (see Figure 2B).

### 3.3. Clinical Features of Patients with ANO3 Variants

The demographic and clinical characteristics of the 17 individuals carrying *ANO3* variants are summarized in Table 1. Among the 16 patients with dystonia, the mean age at onset was 28.19 ± 15.22 years, ranging from 2 to 52 years. Ten patients were male and six were female. The distribution of age at onset was broad, with onset occurring from infancy or childhood to late adulthood.

At onset, most patients presented with focal dystonia, while a smaller proportion presented with segmental or multifocal involvement. The most common initial sites were the neck, upper limbs, and lower limbs. During the disease course, cervical involvement was the most frequent manifestation, observed in 10 of 16 patients, followed by upper limb involvement in eight patients and lower limb involvement in seven patients. Four patients developed generalized dystonia, and four had segmental or multifocal dystonia.

Tremulous dystonia was a common feature and was observed in 12 of 16 patients. Five patients reported alleviating maneuvers. Most patients had isolated dystonia. However, additional neurological or neuropsychiatric features were observed in several individuals, including parkinsonism in two patients and psychiatric or behavioral symptoms in two patients. One non-dystonic individual carrying a likely pathogenic *ANO3* variant presented with bilateral involuntary shoulder movements with tic–choreiform characteristics, suggesting that ANO3-related movement disorders may extend beyond classical isolated dystonia.

### 3.4. Phenotypic Distribution and Disease Evolution

**Figure 3:**
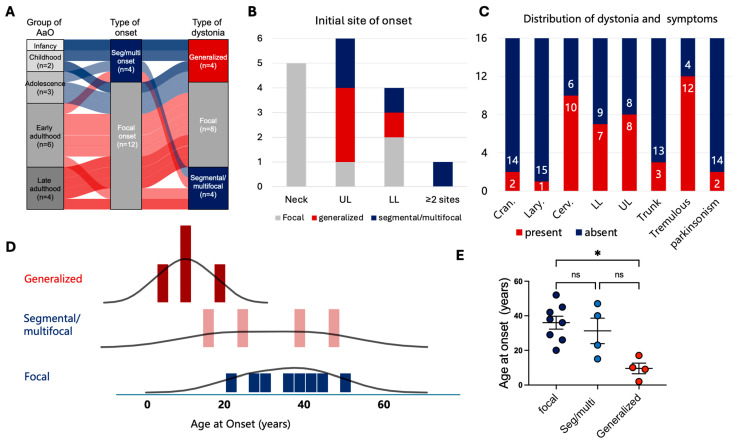
Clinical characteristics of DYT-*ANO3* patients. (**A**) The Sankey diagram illustrates the onset types and progression patterns of DYT-*ANO3* patients across different age groups at onset. Stacked bar charts are employed to illustrate the proportions of different age groups at onset, onset types, and dystonia subtypes. The lines represent the relationship between these three factors for each individual. (**B**) The stacked bar chart illustrates the initial site of onset for carriers of *ANO3* gene variants. Initial sites of involvement are categorized into six distinct groups. (**C**) Distribution of affected sites among dystonia patients carrying *ANO3* gene variants. (**D**) Ridgeline histograms depicting age-at-onset distributions across final dystonia subtypes. Each ridge represents a subtype, and the bars indicate the number of patients at different ages. generalized dystonia showed an earlier age at onset than focal and segmental/multifocal dystonia. (**E**) Kruskal–Wallis test revealed statistically significant differences in AaO between the patient group classified as “generalized” and those classified as distinct types of dystonia. Seg/multi, segmental/multifocal; Cran., cranial; ULs, upper limbs; LLs, lower limbs; Lary., laryngeal; Cerv., cervical; * *p* < 0.05; ns, no significance.

The relationship between age at onset, initial distribution, and final dystonia subtype is illustrated in the Sankey diagram in Figure 3A. This figure provides an overview of the clinical trajectories of patients with ANO3-related dystonia and demonstrates that patients with childhood or adolescent onset tend to show more widespread involvement, whereas in adult-onset patients it more often remains focal or segmental (see Figure 3D,E).

Among the patients with lower limb onset, a higher incidence of progression to generalized dystonia was observed. Conversely, patients with cervical or upper limb onset more frequently maintained a focal or segmental phenotype during the follow-up period. Overall, 6 out of 16 patients exhibited a spread of dystonia to additional body regions, whereas the remaining 10 patients demonstrated a relatively stable anatomical distribution.

The distribution of initial and final affected regions is summarized in Figure 3B,C. Cervical involvement (10/16, 62.5%) was the most common final manifestation, followed by involvement of the upper (8/16, 50%) and lower limbs (7/16, 43.75%). Tremulous dystonia (12/16, 75%) was the most frequently observed accompanying phenomenological feature. Collectively, these findings demonstrate significant clinical heterogeneity in ANO3-related dystonia, spanning a spectrum from adult-onset focal cervical dystonia to early-onset generalized dystonia.

### 3.5. Treatment Outcomes in Patients with ANO3 Mutations

The treatment regimens and responses are summarized in Table 2 and Table S4. Among the dystonia patients with available treatment data, the mean follow-up duration was 39.22 months (range, 2–85 months), and the mean disease duration at the last follow-up was 98.92 months (range, 22.27–303 months).

**Figure 4:**
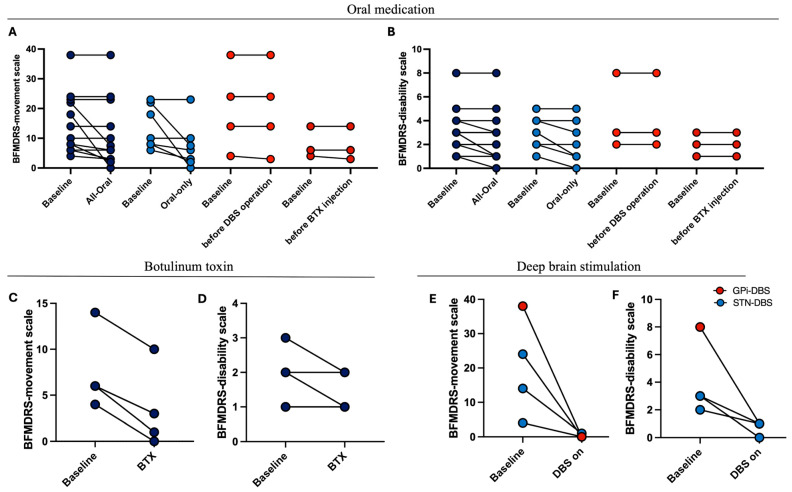
Changes in BFMDRS-M and BFMDRS-D scores for different treatments of DYT-*ANO3* patients. (**A**,**B**) Changes in BFMDRS scores from baseline to follow-up across all patients receiving oral medication. Dark blue represents all patients on oral medication; red indicates preoperative medication response within the DBS treatment subgroup; dark red signifies medication response prior to botulinum toxin injections. Light blue illustrates changes in medication efficacy within the subgroup solely on oral medication. (**C**,**D**) Comparison of BFMDRS score variations between baseline and follow-up prior to and following botulinum toxin administration. (**E**,**F**) Comparison of BFMDRS score alterations from baseline to final follow-up, conducted before and after DBS treatment. Red circles indicate GPi-DBS, while blue circles denote STN-DBS. BFMDRS-D, Burke–Fahn–Marsden Dystonia Rating Scale Disability Score; BFMDRS-M, Burke–Fahn–Marsden Dystonia Rating Scale Movement Score; DBS, deep brain stimulation; GPi, internal globus pallidus; STN, subthalamic nucleus; BTX, botulinum toxin.

Oral medications were used as the initial treatment for most patients. Of 14 patients with available BFMDRS data, the average BFMDRS-M score dropped from 14.50 ± 9.55 at baseline to 10.89 ± 10.86 during follow-up, showing an average improvement of 29.71% ± 36.15. The average BFMDRS-D score shifted from 2.94 ± 1.77 to 2.50 ± 1.97, indicating an average improvement of 22.02% ± 34.22. Overall, the responses to oral medication varied, with significant benefits seen only in some patients (see Figure 4A,B).

A total of five patients received botulinum toxin injections and had available quantitative follow-up data. Within this subgroup, the mean BFMDRS-M score demonstrated an improvement from 7.20 ± 3.90 to 3.40 ± 3.91, corresponding to an average enhancement of 62.38% ± 28.74. The mean BFMDRS-D score changed from 1.80 ± 0.84 to 1.40 ± 0.55, with an average improvement of 16.67% ± 23.57. Clinically, botulinum toxin appeared to be more advantageous for patients suffering from focal dystonia, particularly those with cervical or craniofacial involvement (see Figure 4C,D).

Four patients underwent DBS and had complete quantitative follow-up data, including three treated with STN-DBS and one with GPi-DBS. The mean BFMDRS-M score improved from 20.00 ± 14.51 at baseline to 0.25 ± 0.50 at follow-up, representing a mean improvement of 98.22% ± 3.57. The mean BFMDRS-D score improved from 4.00 ± 2.71 to 0.75 ± 0.50, indicating a mean improvement of 76.04% ± 22.15. All the patients who received DBS exhibited significant clinical improvement during the follow-up period. Due to the limited sample size, no formal statistical comparison between the stimulation targets was conducted (see Figure 4E,F).

Overall, oral medication showed variable benefit, botulinum toxin was useful mainly for focal manifestations, and DBS was associated with substantial improvement in selected patients with medically refractory ANO3-related dystonia.

## 4. Discussion

In this multicenter Chinese cohort, we identified 15 rare *ANO3* missense variants in 16 dystonia patients and one non-dystonic patient after whole-exome sequencing and ACMG filtering. These variants were significantly more common in the dystonia group compared to population datasets and a non-dystonic control group, supporting an association between rare *ANO3* variants and dystonia at the gene level. Several previously unreported variants, including one pathogenic variant and several likely pathogenic candidate variants, expand the potential genetic spectrum of *ANO3* in Chinese patients. The in silico predictions suggest predicted deleteriousness and mechanistic heterogeneity among these variants, though functional validation is needed. Clinically, ANO3-related dystonia showed diverse presentations, from adult-onset focal or segmental to early-onset generalized dystonia, often with tremors. The treatments varied; medications and botulinum toxin helped some, while DBS showed marked improvement in a few refractory cases. Due to the small sample size, especially for DBS, these findings are preliminary and need validation in larger cohorts with standardized follow-up.

### 4.1. Genetic Spectrum of ANO3 and Interpretation of Rare Variants

A total of 15 *ANO3* variants were identified, with one pathogenic de novo variant and five variants classified as likely pathogenic (LP), alongside the majority classified as variants of uncertain significance (VUSs). This distribution is consistent with other recently characterized neurogenetic disorders identified through large-scale sequencing efforts where VUSs are predominant [28,29]. Nonetheless, multiple converging lines of evidence support the potential clinical relevance of these variants: extremely low or absent frequencies in population databases, enrichment from aggregate burden analysis, phenotypic overlap with established DYT-*ANO3* characteristics, identification of one de novo variant, and predicted deleteriousness generated by multiple in silico tools. Collectively, these findings provide supportive evidence for prioritizing *ANO3* VUSs for future functional investigations and extend the *ANO3* variant spectrum in Chinese patients.

The in silico results also provided supportive but not definitive evidence for variant prioritization. All the included variants had CADD scores above 15, and several were predicted to be deleterious by multiple computational tools. The LOGOFunc analysis further suggested that different *ANO3* missense variants may be associated with distinct predicted functional consequences, including possible gain-of-function, loss-of-function, or neutral effects. However, these predictions should be interpreted cautiously and should not be considered equivalent to functional validation. Therefore, while our findings expand the *ANO3* variant spectrum in Chinese patients and support a gene-level association with dystonia, the pathogenicity of individual VUSs remains unresolved and requires further segregation and experimental studies.

### 4.2. Clinical Heterogeneity of ANO3-Related Dystonia

Clinically, ANO3-associated dystonia in our cohort primarily manifested as focal or segmental dystonia affecting the craniofacial and cervical regions, consistent with prior reports [5,9,30]. Importantly, we observed a high prevalence of additional characteristics, including tremulous dystonia and alleviating maneuvers. The identification of a non-dystonic *ANO3* variant carrier presenting primarily with tics and choreiform movements further reinforces the significant phenotypic heterogeneity and suggests that ANO3-related pathology extends beyond isolated dystonia. In addition to focal and segmental dystonia, several patients developed generalized dystonia, and a small number had parkinsonism or psychiatric/behavioral features.

Previous studies have suggested that ANO3-related disease may encompass a broader spectrum of hyperkinetic movement disorders beyond classical dystonia. Reported manifestations include chorea, choreiform movements, hyperkinesias, stereotypies, and complex motor or vocal tics, with some patients presenting with Huntington disease-like or chorea-dominant phenotypes that may later evolve into dystonia [31,32,33,34]. These observations are consistent with the prominent expression of *ANO3* in striatal regions and support the notion of broader basal ganglia circuit dysfunction. In our cohort, a non-dystonic individual carrying the likely pathogenic p.P443L variant exhibited tic–choreiform shoulder movements. Together with previous reports, this observation raises the possibility that ANO3-related movement disorders may encompass a wider phenotypic spectrum, although further clinical and functional studies are needed.

Despite the limited size of the cohort, the Sankey diagram suggests possible relationships between age at onset, initial anatomical involvement, and the subsequent distribution of dystonia. The patients who developed generalized dystonia tended to have a younger age at onset compared to those with focal, segmental, or multifocal forms of the disease. Additionally, onset in the lower limbs appeared to be more frequently associated with a wider anatomical spread, whereas onset in the cervical region or upper limbs appeared more likely to remain relatively restricted during follow-up. These findings should be viewed as exploratory and hypothesis generating, given the small sample size and retrospective design, and they warrant validation in larger longitudinal studies.

Furthermore, the occurrence of asymptomatic *ANO3* variant carriers within multiple families indicates incomplete penetrance of ANO3-associated dystonia, akin to that seen in other DYT-related genes, such as *TOR1A*, *THAP1*, and *VPS16* [35,36,37]. However, our current cohort does not facilitate a definitive estimation of penetrance. The limited number of relatives who have undergone genetic testing, coupled with the incomplete clinical evaluation of extended family members, may result in inaccurate estimations of penetrance. Consequently, future comprehensive segregation studies and longitudinal assessments of *ANO3* variant carriers will be imperative to elucidate the pattern of penetrance and phenotypic variability associated with DYT-*ANO3*.

### 4.3. Therapeutic Implications and Efficacy

Pharmacological treatments offered limited benefit to most patients, while botulinum toxin was primarily effective for focal symptoms, consistent with previous findings [38,39,40]. DBS was associated with marked improvement in selected patients with medically refractory ANO3-related dystonia. Among the four patients with available follow-up data, three underwent STN-DBS and one underwent GPi-DBS, and all showed substantial reductions in both BFMDRS-M and BFMDRS-D scores during follow-up. However, given the very small number of surgically treated patients and the retrospective nature of outcome assessment, these findings should be interpreted as preliminary clinical observations rather than definitive evidence of DBS efficacy.

Previous reports of DBS in DYT-*ANO3* have mainly involved GPi-DBS, whereas STN-DBS has not, to our knowledge, been previously reported in DYT-*ANO3* [13,18,19,20,41,42,43,44,45]. Therefore, our findings provide preliminary clinical evidence suggesting that STN-DBS may be a feasible therapeutic option for selected patients with medication-refractory ANO3-related dystonia.

Given the increased expression of *ANO3* in striatal MSNs and the role of the indirect pathway in shaping basal ganglia output, targeting STN offers a biologically plausible therapeutic option for ANO3-related dystonia (Figure S3). Our findings indicate that both GPi and STN may serve as potential DBS targets for medication-refractory DYT-*ANO3*; however, due to the limited number of patients who underwent surgical treatment, no definitive conclusions can be established. Further validation through meticulously designed prospective randomized controlled studies is essential.

### 4.4. Limitations

This study has limitations. The control group was not fully matched to the dystonia group, and comparisons with external databases may be biased by differences in ancestry and sequencing methods. Most *ANO3* variants were classified as VUS or likely pathogenic without functional tests. Although WES and Sanger sequencing were done, additional genetic tests were not routinely available, limiting exclusion of other genetic causes. Limited information from unaffected relatives hindered assessment of penetrance and expressivity. The treatment outcomes should be interpreted cautiously due to the retrospective design; non-standardized protocols; non-blinded BFMDRS assessments and only four patients who underwent DBS, with incomplete perioperative data in some cases. The DBS results are preliminary. Future prospective multicenter studies with standardized clinical and genetic evaluations are needed to clarify genotype–phenotype links and improve treatment for ANO3-related dystonia.

## 5. Conclusions

This study enhances understanding of ANO3 movement disorders in a Chinese cohort, identifying one pathogenic and five likely pathogenic variants. ANO3-related dystonia presents with diverse clinical features, often focal or segmental with tremors, but can also be generalized or hyperkinetic. Deep brain stimulation, particularly STN-DBS, shows promise for refractory DYT-*ANO3* cases, though larger studies are needed to confirm its efficacy and optimal targets.

## Figures and Tables

**Table 1:** Clinical data of the patients with *ANO3* variants.

Patient No.	Nucleotide, Amino Acid	AoO (Age/Gender)	Inheritance	Initial Distribution	Final Distribution	Body Regions Involved	Tremulous	Alleviating Maneuvers	Associated Features	ACMG	
										Classification	Evidence
Case 1	c.182A > C, p.E61A	45/F	UK	Focal	Focal	LL			Parkinsonism	VUS	PM1 PM2 PP2 BP6
Case 2	c.182A > C, p.E61A	29/F	UK	Focal	Focal	UL	✓		Isolated	VUS	PM1 PM2 PP2 BP6
Case 3	c.730A > G, p.M244V	42/M	UK	Focal	Focal	Neck	✓	✓	Compulsive behavior, irritability	VUS	PM2 PP2
Case 4	c.860G > A, p.R287Q	36/F	UK	Focal	Focal	Neck	✓	✓	Isolated	VUS	PM2 PP2 PP3
Case 5	c.982C > T, p.R328C	52/F	UK	Focal	Focal	Neck	✓	✓	Isolated	VUS	PP2 BP4
Case 6	c.982C > T, p.R328C	17/M	Familial	Focal	Generalized	LF, ULs, trunk, LL	✓		Isolated	VUS	PP2 BP4
Case 7	c.1127A > G, p.Y376C	2/M	Paternal	Multifocal	Generalized	Larynx, neck, ULs, trunk, LLs	✓		Isolated	VUS	PM2 PP2 PP3
Case 8	c.1202G > T, p.W401L	15/F	Paternal	Segmental	Segmental	LF, neck, UL	✓	✓	Pain, mental disorder	LP	PM1 PM2 PP2 PP3
Case 9	c.1577T > C, p.I526T	40/M	UK	Focal	Segmental	Neck, ULs			Isolated	VUS	PM1 PM2 PP2 BP4
Case 10	c.1712A > G, p.Y571C	38/M	UK	Focal	Focal	Neck	✓	✓	Isolated	LP	PM1 PM2 PP2 PP3
Case 11	c.1943A > G, p.N648S	26/F	De novo	Focal	Focal	Neck	✓		Parkinsonism	P	PS2 PM1 PM2 PP2 PP3
Case 12	c.1955C > G, p.S652C	23/M	Paternal	Multifocal	Focal	UL	✓		Isolated	LP	PM1 PM2 PP2 PP3
Case 13	c.2189A > C, p.H730P	20/M	Maternal	Focal	Focal	LL			Isolated	VUS	PM1 PM2 PP2
Case 14	c.2426T > C, p.I809T	47/M	UK	Focal	Multifocal	Neck, LL	✓		Isolated	LP	PM1 PM2 PP2 PP3
Case 15	c.2549G > A, p.C850Y	9/M	Maternal	Focal	Generalized	ULs, LL			Isolated	LP	PM1 PM2 PP2 PP3
Case 16	c.2828A > G, p.H943R	10/M	Maternal	Multifocal	Generalized	ULs, LLs, trunk, neck	✓		Isolated	VUS	PM1 PM2 PP2 BP4
Case 17	c.1328C > T, p.P443L	-/M	UK	N/A	N/A	Neck	N/A	N/A	Tics and chorea	LP	PM1 PM2 PP2 PP3

Abbreviations: ACMG, ACMG Standards and Guidelines for the Interpretation of Sequence Variants; VUS, variant of uncertain significance; LP, likely pathogenic; F, female; M, male; LF, lower face (includes jaw, mouth, and tongue); neck (includes shoulders); UL(s), upper limb(s) (incudes hands but not shoulders); trunk; LL(s), lower limb(s) (includes feet). N/A, not available; UK, unknown.

**Table 2:** Treatment modality and improvement in the 16 dystonic patients with *ANO3* Variants.

Treatment Modality	n	BFMDRS-Motor Part			BFMDRS-Disability Part		
		Baseline (Mean ± SD)	Post (Mean ± SD)	Improvement % (Mean ± SD)	Baseline (Mean ± SD)	Post Score (Mean ± SD)	Improvement % (Mean ± SD)
*Oral medication* ^#^	14	14.50 ± 9.55	10.89 ± 10.86	29.71 ± 36.15	2.94 ± 1.77	2.50 ± 1.97	22.02 ± 34.22
Oral medication only	8	12.88 ± 6.96	6.69 ± 7.37	48.86 ± 37.07	3.00 ± 1.31	2.13 ± 1.73	38.54 ± 39.04
*BTX*	5	7.20 ± 3.90	3.40 ± 3.91	62.38 ± 28.74	1.80 ± 0.84	1.40 ± 0.55	16.67 ± 23.57
*DBS*	4	20.00 ± 14.51	0.25 ± 0.50	98.22 ± 3.57	4.00 ± 2.71	0.75 ± 0.50	76.04 ± 22.15
STN-DBS	3	14.00 ± 10.00	0.33 ± 0.58	97.62 ± 4.12	2.67 ± 0.58	0.67 ± 0.58	72.22 ± 25.46
GPi-DBS	1	38.00	0.00	100.00	8.00	1.00	87.50

Abbreviations: BFMDRS, Burke–Fahn–Marsden Dystonia Rating Scale; BTX, botulinum toxin; DBS, deep brain stimulation; STN, subthalamic nucleus; GPi, globus pallidus internus. ^#^ Treatment outcomes for two patients in the oral medication group are missing.

## Data Availability

The data that support the findings of this study are available from the corresponding author upon reasonable request.

## Supplementary Materials

The following supporting information can be downloaded at: https://www.mdpi.com/article/10.3390/genes17060703/s1, Figure S1: Pedigrees of the cases with *ANO3* variants; Figure S2: Genetic data of the cases with *ANO3* variants; Figure S3: Spatio-temporal distribution patterns and single-cell expression distribution characteristics of the *ANO3* gene; Table S1: Diagnostic composition of the non-dystonic movement disorder control cohort; Table S2: In silico pathogenicity and predicted functional mechanisms of rare *ANO3* missense variants; Table S3: Aggregate frequency of *ANO3* variants; Table S4: Summary of clinical follow-up, treatment regimens, and therapeutic responses in patients with *ANO3* variants; Table S5: Detailed variant-level information, inclusion/exclusion criteria, and secondary WES findings for individuals carrying *ANO3* variants.

## References

[1] Albanese A., Bhatia K.P., Fung V.S.C., Hallett M., Jankovic J., Klein C., Krauss J.K., Lang A.E., Mink J.W., Pandey S. (2025). Definition and Classification of Dystonia. Mov. Disord..

[2] Albanese A., Bhatia K., Bressman S.B., Delong M.R., Fahn S., Fung V.S., Hallett M., Jankovic J., Jinnah H.A., Klein C. (2013). Phenomenology and classification of dystonia: A consensus update. Mov. Disord..

[3] Pozojevic J., Beetz C., Westenberger A. (2021). The importance of genetic testing for dystonia patients and translational research. J. Neural Transm..

[4] Charlesworth G., Plagnol V., Holmström K.M., Bras J., Sheerin U.M., Preza E., Rubio-Agusti I., Ryten M., Schneider S.A., Stamelou M. (2012). Mutations in ANO3 cause dominant craniocervical dystonia: Ion channel implicated in pathogenesis. Am. J. Hum. Genet..

[5] Jiang L.T., Li L.X., Liu Y., Zhang X.L., Pan Y.G., Wang L., Wan X.H., Jin L.J. (2021). The expanding clinical and genetic spectrum of ANO3 dystonia. Neurosci. Lett..

[6] Li L.X., Liu Y., Huang J.H., Yang Y., Pan Y.G., Zhang X.L., Pan L.Z., Jin L.J. (2023). Genetic spectrum and clinical features in a cohort of Chinese patients with isolated dystonia. Clin. Genet..

[7] Stamelou M., Charlesworth G., Cordivari C., Schneider S.A., Kägi G., Sheerin U.M., Rubio-Agusti I., Batla A., Houlden H., Wood N.W. (2014). The phenotypic spectrum of DYT24 due to ANO3 mutations. Mov. Disord..

[8] Lange L.M., Junker J., Loens S., Baumann H., Olschewski L., Schaake S., Madoev H., Petkovic S., Kuhnke N., Kasten M. (2021). Genotype-Phenotype Relations for Isolated Dystonia Genes: MDSGene Systematic Review. Mov. Disord..

[9] Percetti M., Zini M., Soliveri P., Cogiamanian F., Ferrara M., Orunesu E., Ranghetti A., Ferrarese C., Pezzoli G., Garavaglia B. (2024). The Clinical Spectrum of ANO3-Report of a New Family and Literature Review. Mov. Disord. Clin. Pract..

[10] Ma L.Y., Wang L., Yang Y.M., Feng T., Wan X.H. (2015). Mutations in ANO3 and GNAL gene in thirty-three isolated dystonia families. Mov. Disord..

[11] Yoo H.S., Lee H., Chung S.J., Lee J.S., Hong S.K., Lee P.H., Kim Y.J., Sohn Y.H., Shin H.W. (2018). A Novel Heterozygous ANO3 Mutation with Basal Ganglia Dysfunction in a Patient with Adult-Onset Isolated Segmental Dystonia. J. Clin. Neurol..

[12] Kuo M.C., Lin H.I., Lin C.H. (2019). Craniocervical dystonia with levodopa-responsive parkinsonism co-segregating with a pathogenic ANO3 mutation in a Taiwanese family. Park. Relat. Disord..

[13] Li S., Wang L., Yang Y., Ma J., Wan X. (2019). ANO3 Mutations in Chinese Dystonia: A Genetic Screening Study Using Next-Generation Sequencing. Front. Neurol..

[14] Wu M.C., Chang Y.Y., Lan M.Y., Chen Y.F., Tai C.H., Lin Y.F., Tsai S.F., Chen P.L., Lin C.H. (2022). A Clinical and Integrated Genetic Study of Isolated and Combined Dystonia in Taiwan. J. Mol. Diagn..

[15] Ma J., Wang L., Yang Y.M., Wan X.H. (2018). Targeted gene capture sequencing in diagnosis of dystonia patients. J. Neurol. Sci..

[16] Fu F., Kang Y., Li J., Jin N., Zheng X., Cen Z., Luo W. (2024). A Novel ANO3 Gene Mutation Associated with a Dystonia-Ataxia Syndrome. Mov. Disord. Clin. Pract..

[17] Saini A., Singh I., Kumar M., Radhakrishnan D.M., Agarwal A., Garg D., Elavarasi A., Singh R., Chouhan V., Sandeep (2025). Genetic Landscape of Dystonia in Asian Indians. Mov. Disord. Clin. Pract..

[18] Zech M., Boesch S., Jochim A., Weber S., Meindl T., Schormair B., Wieland T., Lunetta C., Sansone V., Messner M. (2017). Clinical exome sequencing in early-onset generalized dystonia and large-scale resequencing follow-up. Mov. Disord..

[19] Yoo D., Kim H.J., Chae J.H., Paek S.H., Jeon B. (2019). Successful Pallidal Deep Brain Stimulation in a Patient with Childhood-Onset Generalized Dystonia with ANO3 Mutation. J. Mov. Disord..

[20] Olschewski L., Jesús S., Kim H.J., Tunc S., Löns S., Junker J., Zeuner K.E., Kühn A.A., Kuhlenbäumer G., Schäffer E. (2019). Role of ANO3 mutations in dystonia: A large-scale mutational screening study. Park. Relat. Disord..

[21] Chen S., Francioli L.C., Goodrich J.K., Collins R.L., Kanai M., Wang Q., Alfoldi J., Watts N.A., Vittal C., Gauthier L.D. (2024). A genomic mutational constraint map using variation in 76,156 human genomes. Nature.

[22] Hao M., Pu W., Li Y., Wen S., Sun C., Ma Y., Zheng H., Chen X., Tan J., Zhang G. (2021). The HuaBiao project: Whole-exome sequencing of 5000 Han Chinese individuals. J. Genet. Genom..

[23] Richards S., Aziz N., Bale S., Bick D., Das S., Gastier-Foster J., Grody W.W., Hegde M., Lyon E., Spector E. (2015). Standards and guidelines for the interpretation of sequence variants: A joint consensus recommendation of the American College of Medical Genetics and Genomics and the Association for Molecular Pathology. Genet. Med..

[24] Li Q., Wang K. (2017). InterVar: Clinical Interpretation of Genetic Variants by the 2015 ACMG-AMP Guidelines. Am. J. Hum. Genet..

[25] Wang Z., Zhao G., Zhu Z., Wang Y., Xiang X., Zhang S., Luo T., Zhou Q., Qiu J., Tang B. (2024). VarCards2: An integrated genetic and clinical database for ACMG-AMP variant-interpretation guidelines in the human whole genome. Nucleic Acids Res..

[26] CONVERGE Consortium (2015). Sparse whole-genome sequencing identifies two loci for major depressive disorder. Nature.

[27] Burke R.E., Fahn S., Marsden C.D., Bressman S.B., Moskowitz C., Friedman J. (1985). Validity and reliability of a rating scale for the primary torsion dystonias. Neurology.

[28] Burke W., Parens E., Chung W.K., Berger S.M., Appelbaum P.S. (2022). The Challenge of Genetic Variants of Uncertain Clinical Significance: A Narrative Review. Ann. Intern. Med..

[29] Walsh R., Lahrouchi N., Tadros R., Kyndt F., Glinge C., Postema P.G., Amin A.S., Nannenberg E.A., Ware J.S., Whiffin N. (2021). Enhancing rare variant interpretation in inherited arrhythmias through quantitative analysis of consortium disease cohorts and population controls. Genet. Med..

[30] Balint B., Bhatia K.P. (2014). Dystonia: An update on phenomenology, classification, pathogenesis and treatment. Curr. Opin. Neurol..

[31] Blackburn P.R., Zimmermann M.T., Gass J.M., Harris K.G., Cousin M.A., Boczek N.J., Ross O.A., Klee E.W., Brazis P.W., Van Gerpen J.A. (2016). A novel ANO3 variant identified in a 53-year-old woman presenting with hyperkinetic dysarthria, blepharospasm, hyperkinesias, and complex motor tics. BMC Med. Genet..

[32] Koya Kutty S., Mulroy E., Magrinelli F., Di Lazzaro G., Latorre A., Bhatia K.P. (2021). Huntington disease-like phenotype in a patient with ANO3 mutation. Park. Relat. Disord..

[33] Romito L.M., Leta V., Garavaglia B., Panteghini C., Zorzi G., Elia A.E., Colucci F., Carecchio M., Eleopra R. (2024). ANO3 as a Cause of Early-Onset Chorea Combined with Dystonia: Illustration of Phenotypic Evolution. Mov. Disord..

[34] Akcimen F., Diez-Fairen M., Alvarez I., Puente V., Grant S., Hernandez-Vara J., Khani M., Buongiorno M., Jimenez-Jimenez F.J., Agundez J.A.G. (2026). Unraveling the genetic architecture of non-Huntington chorea: A biobank-scale study of rare variants and repeat expansions. npj Genom. Med..

[35] Eidelberg D., Moeller J.R., Antonini A., Kazumata K., Nakamura T., Dhawan V., Spetsieris P., deLeon D., Bressman S.B., Fahn S. (1998). Functional brain networks in DYT1 dystonia. Ann. Neurol..

[36] Houlden H., Schneider S.A., Paudel R., Melchers A., Schwingenschuh P., Edwards M., Hardy J., Bhatia K.P. (2010). THAP1 mutations (DYT6) are an additional cause of early-onset dystonia. Neurology.

[37] Westenberger A., Verdura E., Radefeldt M., Sanderson L.E., Tripolszki K., Marce-Grau A., Cazurro-Gutierrez A., Nikoncuk A., Herzog R., Al-Ali R. (2026). Expanding the Genetic and Phenotypic Spectrum of DYT-VPS16: The Importance of Splice-Site Variants. Mov. Disord..

[38] Balash Y., Giladi N. (2004). Efficacy of pharmacological treatment of dystonia: Evidence-based review including meta-analysis of the effect of botulinum toxin and other cure options. Eur. J. Neurol..

[39] Kaplan E.H., Vecchio M., Simpson D.M. (2025). Review: Botulinum Toxin for Treatment of Focal Limb Dystonia. Toxins.

[40] Zoons E., Dijkgraaf M.G., Dijk J.M., van Schaik I.N., Tijssen M.A. (2012). Botulinum toxin as treatment for focal dystonia: A systematic review of the pharmaco-therapeutic and pharmaco-economic value. J. Neurol..

[41] Boddu A.V., Brinkerhoff S., Bashir A.E., Crowder C.M., Awad M., Gonzalez C.L., Walker H.C. (2024). Directional Stimulus-Evoked Pallidal Electrophysiology in Primary Dystonia. Tremor Other Hyperkinet. Mov..

[42] Di Luca D.G., Grippe T.C., Adams J., Chen R., Fasano A., Lozano A., Lang A.E. (2024). Generalized Dystonia With Tremor and Myoclonus Associated With ANO3 Variant. Can. J. Neurol. Sci..

[43] Lasky L., Bliss L., Sidiropoulos C. (2019). Successful Pallidal Deep Brain Stimulation Treatment in a Case of Generalized Dystonia due to a Novel ANO3 Mutation. Case Rep. Neurol. Med..

[44] Poulen G., Chan-Seng E., Sanrey E., Coubes P. (2024). A Case of Successful Pallidal Deep Brain Stimulation in ANO3 Dystonia. Mov. Disord..

[45] Delamarre A., Chelly J., Guehl D., Drouot N., Tranchant C., Anheim M., Burbaud P. (2019). Novel anoctamin-3 missense mutation responsible for early-onset myoclonic dystonia. Park. Relat. Disord..

