# Supplementary material for: Clinical and Genetic Spectrum of ANO3-Related Dystonia with Treatment Responses in a Chinese Cohort
Source: Genes (Basel). 2026 Jun 17;17(6):703. doi: 10.3390/genes17060703 (PMC13299146; doi:10.3390/genes17060703)
Supplement: Supplementary file 1 [file genes-17-00703-s001.zip › genes-4312598-supplementary file 1.pdf]

Supplementary materials file 1

Title: Clinical and genetic spectrum of ANO3-related dystonia with treatment responses in a Chinese cohort

Jie-Hong Huang [1,3 #], Yang Li [2 #], Zhi-Meng Wan [2], Li-Xi Li [3], Ling-Jing Jin [1,3,4*], Xin-Hua Wan [2 *]

[1] Department of Neurology and Neurological Rehabilitation, Shanghai Disabled Persons' Federation Key Laboratory of Intelligent Rehabilitation Assistive Devices and Technologies, Yangzhi Rehabilitation Hospital (Shanghai Sunshine Rehabilitation Center), School of Medicine, Tongji University, Shanghai, China

[2] Department of Neurology, Peking Union Medical College Hospital, Chinese Academy of Medical Sciences, Peking Union Medical College, Beijing, China

[3] Neurotoxin Research Center of Key Laboratory of Spine and Spinal Cord Injury Repair and Regeneration of Ministry of Education, Department of Neurology, Tongji Hospital, School of Medicine, Tongji University, Shanghai, China.

[4] Collaborative Innovation Center for Brain Science, Tongji University, Shanghai, China.

[#] These two authors contributed equally to this work.

[*] **Corresponding author:**

Prof. Xin-Hua Wan, E-mail address: wxhpumch@163.com.

Prof. Ling-Jing Jin, E-mail address: lingjingjin@163.com.

Running title: ANO3-related dystonia

Keywords: ANO3, dystonia, whole-exome sequencing

Supplemental Methods

Whole-exome sequencing

Collect 4 ml of venous blood from the individual into an Ethylenediaminetetraacetic acid (EDTA) anticoagulated tube. Extract genomic DNA utilizing FlexiGene DNA kits (Qiagen, product number 51206). The DNA concentration was measured with an ultramicro spectrophotometer, the Nanodrop 2000 (Thermo Fisher Scientific), employing Agilent SureSelect XT Human All Exon V6 (Agilent). DNA was fragmented into 150-300 base pair fragments using an ultrasonicator (Covaris S2, USA). Subsequently, the DNA fragments underwent processing, including end-repair, A-tailing, adaptor ligation, PCR amplification, and enrichment using the whole-exome panel. Paired-end sequencing was performed on the Illumina HiSeq X-ten platform, achieving an average sequence coverage exceeding 100X and at least 20X coverage in more than 95% of target regions. The resulting data were processed using bioinformatics tools.

For quality control purposes, a few unqualified sequences from the primary data, including 3′-/5′- adaptor sequences and low-quality reads, were removed utilizing Cutadapt and FastQC, respectively. The remaining sequences were designated as clean reads for subsequent analysis. These clean reads were then aligned to the reference human genome (UCSC hg19) employing BWA (Burrows Wheeler Aligner version 0.7.12-r1039). Duplicates were marked and eliminated using Picard Tools following default parameters. Short read alignment and variant visualization were conducted with the IGV (Integrative Genomics Viewer). SNVs and indels were identified through GATK (The Genome Analysis Toolkit version 3.7). Further annotation was performed via ANNOVAR, incorporating various descriptive data, including functional implications (such as gene region, functional effect, mRNA GenBank accession number, amino acid alteration, etc.) and allele frequencies in population databases (gnomAD-all, gnomAD-EAS, and HUABIAO databases).

Spatiotemporal and single-cell expression analyses of *ANO3*

Publicly available bulk and single-cell transcriptomic datasets, including the Human Protein Atlas (https://www.proteinatlas.org/), GTEx, BrainSpan (https://www.brainspan.org/), and the Human Brain Cell Atlas(https://cellxgene.cziscience.com), were examined to assess the regional, developmental, and cell-type–specific expression patterns of *ANO3*. These analyses aimed to provide biological context for the clinical heterogeneity observed within our cohort. A detailed visualization of these expression profiles is provided in Fig. S3.

Figure S1: Pedigrees of the cases with *ANO3* variants.

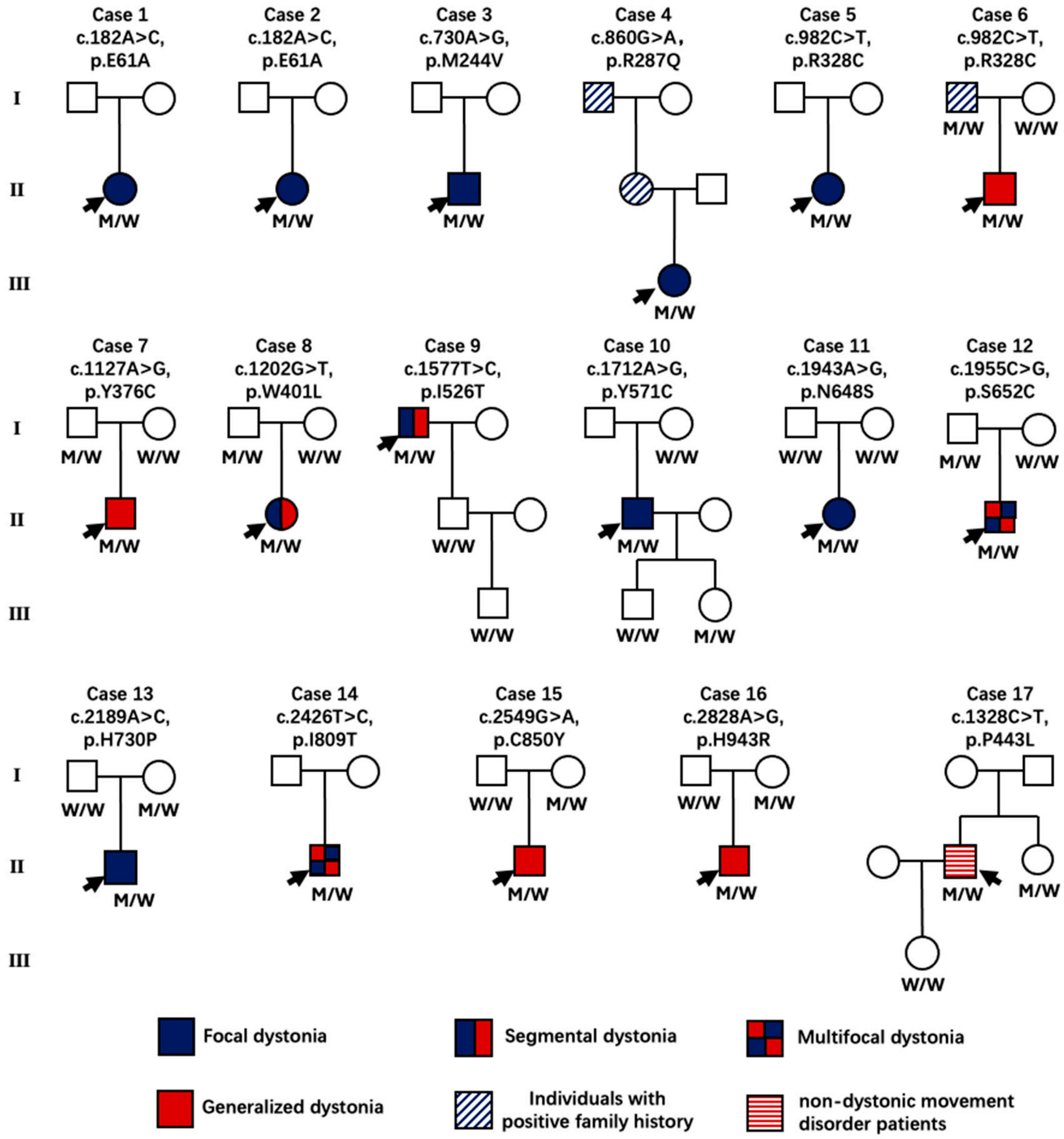

Pedigrees and their corresponding phenotypes of the 17 cases with *ANO3* variants. M/W indicated individuals with variants, and those without variants were indicated by W/W. The proband is indicated by the black arrow.

Figure S2: Genetic data of the cases with *ANO3* variants.

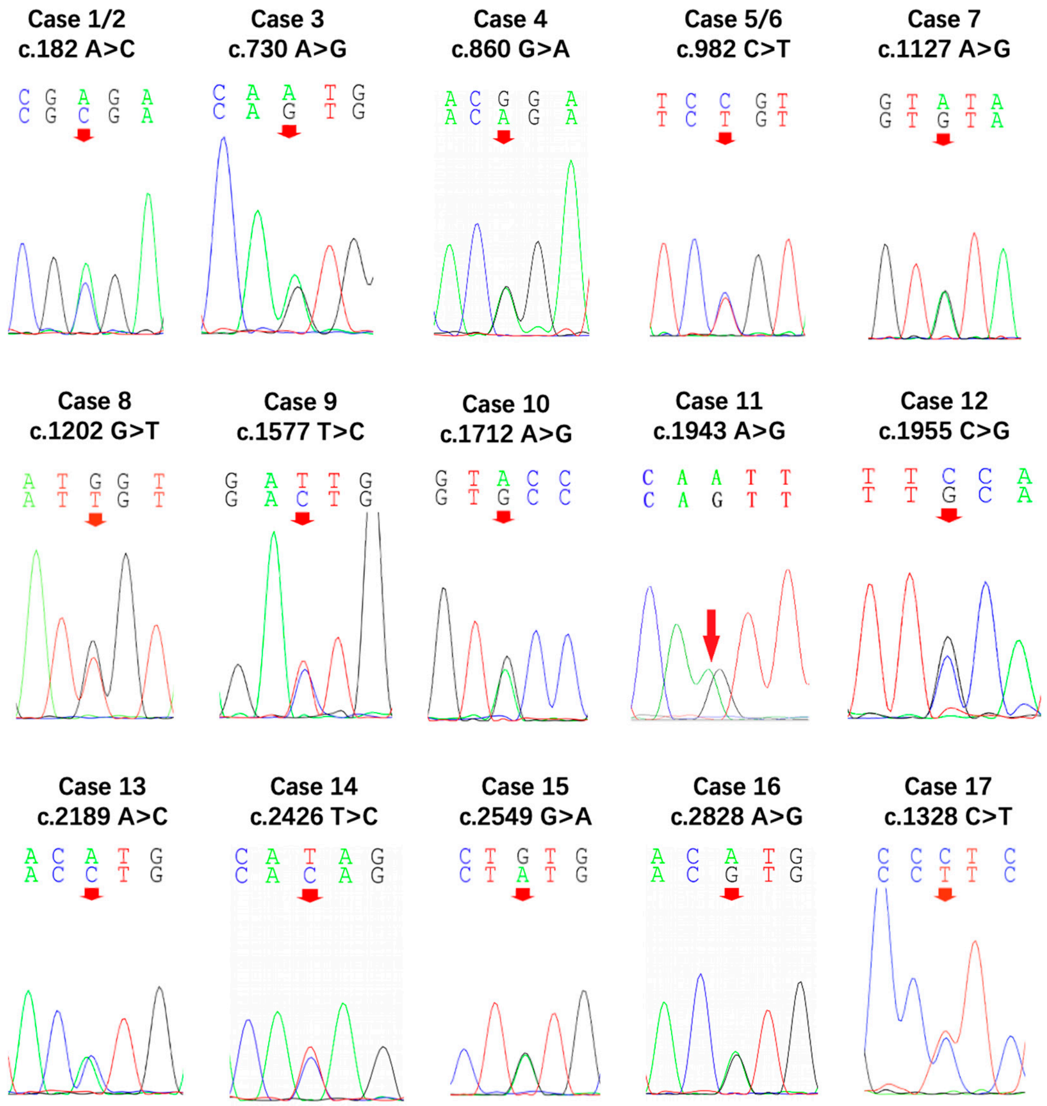

DNA sequencing chromatograms of *ANO3* variants.

Figure S3: Spatio-temporal distribution patterns and single-cell expression distribution characteristics of the *ANO3* gene.

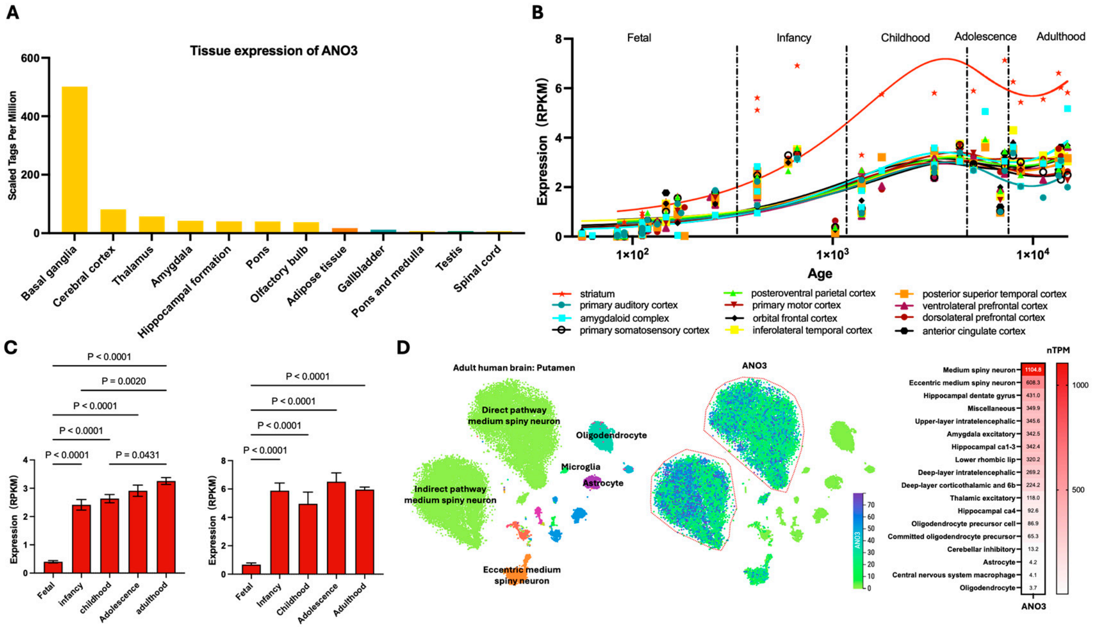

(A) The *ANO3* expression levels of the top 12 tissues were visualized. ANO3 showed relatively high expression in the basal ganglia. The RNA expression was analyzed by the data of the Human Protein Atlas (HPA) dataset. TPM: Transcripts per kilobase per million mapped reads. (B) Curves and dots in different colors represented the temporal expression pattern of *ANO3* in 12 brain areas. The human RNA expression levels were retrieved from the BrainSpan database. The curves were fitted by the locally weighted scatterplot smoothing (LOWESS) algorithm. RPKM, reads per kilobase per million mapped reads. (C) One-way ANOVA analysis revealed statistically significant differences in ANO3 expression during both foetal development and distinct postnatal periods. Left: data from 12 brain areas; Right: data from Striatum (D) The single-cell expression data of *ANO3* was retrieved from the putamen of adult donor brains. The Human Proteome Atlas (HPA) employed raw data from the Human Brain Cell Atlas v1.0 dataset to analyse RNA expression patterns across distinct cell types at the single-cell level. TPM: Transcripts per kilobase per million mapped reads.

Table S1: Diagnostic composition of the non-dystonic movement disorder control cohort

Diagnosis category	n	Percentage (%)
**Parkinson's disease and parkinsonism**	**124**	**40.7**
**Essential tremor**	**40**	**13.1**
**Paroxysmal symptoms**	**32**	**10.5**
**Hypoxic-ischemic encephalopathy and cerebral palsy**	**30**	**9.8**
**Tardive dyskinesia**	**24**	**7.9**
**Paroxysmal chorea**	**23**	**7.5**
**Tic disorders**	**18**	**5.9**
**Neuroacanthocytosis**	**6**	**2.0**
**Stiff-person syndrome**	**5**	**1.6**
**Myalgia of unknown cause**	**3**	**1.0**
**Total**	**305**	

Values are shown as n (%). All individuals in the non-DYT cohort were evaluated by experienced movement disorder neurologists and had no clinical evidence of dystonia.

Table S2: *In silico* pathogenicity and predicted functional mechanisms of rare *ANO3* missense variants

Variation nucleotide	NCBI transcript	Genomic Position (GRCh37/hg19)	Pathogenicity reported in	Variation amino acid	GERP++	SIFT	PolyPhen2_HDIV	PolyPhen2_HVAR	Mutation Taster	Mutation Assessor	MetaSVM	CADD	phastCons_primate	SiPhy_Phred	LOGOFunc
c.182A>C	NM_031418.4	11:26463600-A-C	[13]	p.E61A	NC (6.47)	T (0.189)	B (0.255)	B (0.057)	Po (0.140)	N (0.690)	T (-1.048)	**D (19.22)**	NC (4.23)	NC (2.74)	Neutral (0.81)
c.730A>G	NM_031418.4	11:26547219-A-G	Novel	p.M244V	NC (6.10)	T (0.228)	B (0.005)	B (0.027)	Po (0.110)	**L (1.070)**	T (-0.594)	**D (21.40)**	**C (35.77)**	NC (7.21)	Neutral (0.67)
c.860G>A	NM_031418.4	11:26552874-G-A	[11]	p.R287Q	**C (17.66)**	**D (0.005)**	**PD (1.000)**	**PD (0.994)**	Po (0.300)	**M (2.955)**	**D (0.035)**	**D (31.00)**	NC (3.28)	**C (26.84)**	**GOF (0.44)**
c.982C>T	NM_031418.4	11:26558958-C-T	[20]	p.R328C	NC (2.11)	T (0.084)	B (0.000)	B (0.000)	Po (0.100)	**L (1.445)**	T (-0.954)	**D (24.80)**	NC (11.82)	NC (0.14)	Neutral (0.76)
c.1127A>G	NM_031418.4	11:26563588-A-G	[5]	p.Y376C	**C (16.61)**	**D (0.000)**	**PD (1.000)**	**PD (0.999)**	**DC (0.700)**	**M (3.290)**	**D (0.181)**	**D (29.50)**	**C (19.23)**	NC (14.37)	**LOF (0.41)**
c.1202G>T	NM_031418.4	11:26569010-G-T	Novel	p.W401L	**C (18.75)**	**D (0.004)**	**PD (0.756)**	**PD (0.739)**	**DC (0.980)**	**L (1.530)**	T (-0.6937)	**D (31.00)**	**C (19.23)**	**C (30.88)**	Neutral (0.53)
c.1577T>C	NM_031418.4	11:26620451-T-C	Novel	p.I526T	NC (5.94)	T (0.098)	B (0.005)	B (0.014)	Po (0.110)	N (0.425)	T (-1.027)	**D (21.30)**	NC (4.31)	NC (5.00)	Neutral (0.86)
c.1712A>G	NM_031418.4	11:26621137-A-G	Novel	p.Y571C	**C (18.55)**	**D (0.000)**	**PD (0.999)**	**PD (0.976)**	**DC (0.510)**	**M (2.865)**	**D (0.184)**	**D (28.10)**	NC (5.94)	**C (16.30)**	Neutral (0.45)
c.1943A>G	NM_031418.4	11:26655820-A-G	[45]	p.N648S	**C (58.33)**	**D (0.000)**	**PD (1.000)**	**PD (0.999)**	**DC (0.960)**	**M (3.415)**	**D (0.280)**	**D (25.90)**	NC (13.20)	**C (19.45)**	**GOF (0.49)**
c.1955C>G	NM_031418.4	11:26655832-C-G	Novel	p.S652C	**C (58.33)**	**D (0.000)**	**PD (1.000)**	**PD (1.000)**	**DC (0.85)**	**M (3.050)**	**D (0.0209)**	**D (26.90)**	NC (4.48)	**C (66.45)**	**GOF (0.52)**
c.2189A>C	NM_031418.4	11:26663490-A-C	Novel	p.H730P	**C (43.23)**	T (0.283)	B (**0.044**)	B (**0.063)**	Po (**0.19**)	N (0.540)	T (-0.8315)	**D (22.70)**	NC (7.38)	**C (18.94)**	Neutral (0.85)
c.2426T>C	NM_031418.4	11:26664879-T-C	Novel	p.I809T	**C (23.77)**	**D (0.000)**	**PD (1.000)**	**PD (0.999)**	**DC (0.870)**	**H (3.595)**	**D (0.606)**	**D (29.80)**	NC (13.20)	**C (16.50)**	**LOF (0.72)**
c.2549G>A	NM_031418.4	11:26669376-G-A	Novel	p.C850Y	**C (16.61)**	T (0.566)	**PD (0.999)**	**PD (0.985)**	**DC (0.920)**	**L (1.955)**	T (-0.325)	**D (31.00)**	NC (10.31)	**C (26.26)**	Neutral (0.37)
c.2828A>G	NM_031418.4	11:26681873-A-G	Novel	p.H943R	NC (1.85)	T (0.740)	B (0.000)	B (0.001)	Po (0.080)	N (-2.520)	T (-0.968)	**D (18.18)**	NC (7.66)	NC (1.57)	Neutral (0.85)
c.1328C>T	NM_031418.4	11:26574834-C-T	Novel	p.P443L	**C (18.75)**	**D (0.000)**	**PD (1.000)**	**PD (1.000)**	**DC (0.78)**	**H (3.91)**	**D (0.6678)**	**D (29.90)**	**C (24.77)**	**C (30.88)**	**LOF (0.43)**

The damage of the *ANO3* variants was predicted by *in silico* algorithms (https://genemed.tech/varcards2/). Owing to space limitations, only some typical results were indicated in this table. Abbreviations: B, benign; C, conserved; CADD, combined annotation dependent depletion; D, damaging; DC, Disease causing; GERP++, Genomic Evolutionary Rate Profiling; NC, nonconserved; P, pathogenic; PD, Probably damaging; phastCons, Phylogenetic Analysis with Space/Time models conservation scoring and identification of conserved elements; phyloP, Phylogenetic Analysis with Space/Time Models; Po, polymorphism; PoD, Possibly damaging; SIFT, Sorting Intolerant From Tolerant; T, tolerable; LoGoFunc was used to predict the most likely functional consequence of each ANO3 missense variant among neutral, gain-of-function (GOF), and loss-of-function (LOF) categories. The predicted mechanism was assigned according to the category with the highest probability score. Values in parentheses indicate the probability score for the predicted category.

Table S3: Aggregate frequency of *ANO3* variants

Pt No.	Identified Variants	Allele Count/Number in this study	Allele Count/Number in the gnomAD-all population (v4.1.0)	P value	OR (95% CI)	Allele Count /Number in the gnomAD-EAS population (v4.1.0)	P value	OR (95% CI)	Allele Count/Number in the HUABIAO project	P value	OR (95% CI)
**Dystonia**											
1/2	c.182A>C	2/712 (2.81E-03)	108/1614082 (6.69E-05)	**1.13E-03**	42.10(10.37-170.82)	19/44898 (4.23E-04)	**4.20E-02**	6.65(1.55-28.62)	5/9920(5.04E-04)	7.51E-02	5.59(1.08-28.84)
3	c.730A>G	1/712 (1.40E-03)	6/1610222 (3.73E-06)	**3.09E-03**	377.45(45.38-3139.33)	2/44654 (4.48E-05)	**4.63E-02**	31.40(2.84-346.71)	-	6.70E-02	inf(1.548-inf)
4	c.860G>A	1/712 (1.40E-03)	5/1603422 (3.12E-06)	**2.66E-03**	451.03(52.63-3865.58)	0/44124 (0)	**1.59E-02**	inf(6.89-inf)	-	6.70E-02	inf(1.548-inf)
5/6	c.982C>T	2/712 (2.81E-03)	32/1612858 (1.98E-05)	**1.08E-04**	141.97(33.96-593.55)	27/44872 (6.02E-04)	7.50E-02	4.68(1.11-19.71)	12/9920(1.21E-03)	2.40E-01	2.33(0.52-10.41)
7	c.1127A>G	1/712 (1.40E-03)	7/1612506 (4.34E-06)	**3.53E-03**	323.99(39.81-2636.83)	7/44846 (1.56E-04)	1.18E-01	9.01(1.11-73.32)	6/9920(6.05E-04)	3.85E-01	2.32(0.28-19.33)
8	c.1202G>T	1/712 (1.40E-03)	4/1611850(2.48E-06)	**2.21E-03**	566.75(63.27-5077.14)	4/44776(8.93E-05)	7.59E-02	15.74(1.76-141.03)	-	6.70E-02	inf(1.548-inf)
9	c.1577T>C	1/712 (1.40E-03)	6/1613966 (3.72E-06)	**3.08E-03**	378.33(45.49-3146.63)	2/44864 (4.46E-05)	**4.61E-02**	31.55(2.86-348.34)	1/9920(1.01E-04)	1.29E-01	13.95(0.87-223.28)
10	c.1712A>G	1/712 (1.40E-03)	1/1614104 (6.20E-07)	**8.82E-04**	2270.19(141.85-36331.88)	0/44866 (0)	**1.56E-02**	inf(7.00-inf)	-	6.70E-02	inf(1.548-inf)
11	c.1943A>G	1/712 (1.40E-03)	1/1613826 (6.20E-07)	**8.82E-04**	2269.80(141.83-36325.63)	0/44850 (0)	**1.56E-02**	inf(7.00-inf)	-	6.70E-02	inf(1.548-inf)
12	c.1955C>G	1/712 (1.40E-03)	1/1613258 (6.20E-07)	**8.82E-04**	2269.00(141.78-36312.84)	0/44836 (0)	**1.56E-02**	inf(7.00-inf)	-	6.70E-02	inf(1.548-inf)
13	c.2189A>C	1/712 (1.40E-03)	-	**4.41E-04**	6807(277.10-167228.00)	-	**1.56E-02**	inf(7.01-inf)	-	6.70E-02	inf(1.548-inf)
14	c.2426T>C	1/712 (1.40E-03)	-	**4.41E-04**	6807(277.10-167228.00)	-	**1.56E-02**	inf(7.01-inf)	-	6.70E-02	inf(1.548-inf)
15	c.2549G>A	1/712 (1.40E-03)	-	**4.41E-04**	6807(277.10-167228.00)	-	**1.56E-02**	inf(7.01-inf)	-	6.70E-02	inf(1.548-inf)
16	c.2828A>G	1/712 (1.40E-03)	2/1613614 (1.24E-06)	**1.32E-03**	1134.75(102.78-12528.88)	0/44870 (0)	**1.56E-02**	inf(7.00-inf)	-	6.70E-02	inf(1.548-inf)
Total	total	16/712 (2.25E-02)	173/1614324(1.07E-04)	**1.06E-31**	214.49(127.84-359.89)	61/44898(1.36E-03)	**5.96E-14**	16.90(9.70-29.45)	24/9920(2.42E-03)	**1.91E-09**	9.48(5.01-17.93)
**Non-dystonia**											
17	c.1328C>T	1/610 (1.64E-03)	-	**3.78E-04**	7946 (323.4-195242)	-	**1.34E-02**	Inf (8.18-inf)	-	5.79E-02	Inf (1.81-inf)

P-values and odds ratio were estimated with two-sided Fisher's exact test. Abbreviations: CI, confidence interval; gnomAD, Genome Aggregation Database; OR, odd ratio. the NCBI transcript of these variants is NM_031418.

Table S4: Summary of clinical follow-up, treatment regimens, and therapeutic responses in patients with *ANO3* variants.

**Pt No.**	**Duration (up to last follow-up)**	**Follow up**	**Baseline**	**Oral medication**			**BTX**			**DBS**		
			BFMDRS (M/D)	Current treatment regimen	BFMDRS (M/D)	Improvement %	Regimen	BFMDRS (M/D)	Improvement %	Regimen (Age/follow up)	BFMDRS (M/D)	Improvement %
1	10y	7y	8/2	SEL.	6/2	25/0	-	-	-	-	-	-
2	12y5m	2m	6/4	BAC. 1#tid, THP1# bid	3/3	50/25	-	-	-	-	-	-
3	6y7m	1y6m	6/2	-	-	-	100 U/session × 3	3/2	50/0	-	-	-
4	1y11m	0	6/1	SEL. 1# bid, CZP1/4# qn	NA/NA	NA/NA	NA	1/1		-	-	-
5	4y11m	2y4m	4/2	ARI.，CZP，PGB	3/2	25/0	200 U/session × 4	3/2	25/0	STN-DBS (55y/1y)	0/1	100/50
6	10y	5y10m	38/8	THP, BAC, CZP, SEL	38/8	0/0	-	-	-	GPi-DBS (21ys/5y)	0/1	100/87.5
7	21y9m	3y5m	22/2	ZNS 1# bid, CZP1/4qn, OXC. 1/2 qd	7.5/1	65.91/50	-	-	-	-	-	-
8	3y4m	3y	18/1	DTBZ and THP.	0/0	100/100	-	-	-	-	-	-
9	25y3m	5y2m	23/4	L/B 1/4tid was ineffective, L/C1#bid, PPX 0.25mg tid	23/4	0/0	-	-	-	-	-	-
10	1y9m	6m	6/1	THP1# bid, CZP 1/4 #qn,	6/1	0/0	300 U/session × 4	3/1	50/0	-	-	-
11	2y8m	6m	8/3	THP1#bid, L/B 1/2bid, FLU. 20mg qd, CoQ10 1# tid	2/1	75/66.67	-	-	-	-	-	-
12	12y	7y	10/5	TPM 1# bid, CZP 0.5# qn, GBP1# qd, ZPD 1# qn, ARO. 1# bid	10/5	0/0	-	-	-	-	-	-
13	5y4m	2y	8/3	AMA. 1# bid, THP 1# tid, BAC. 1# tid	2/1	75/66.67	-	-	-	-	-	-
14	5y4m	4y9m	14/3	AMA. 1# bid, THP1/2 bid	14/3	0/0	-	-	-	STN-DBS (48ys/4y)	1/1	92.85/66.66
15	3y3m	4m	14/3	THP1#tid, L/B 1/4 qd, BAC. 1/2 tid	14/3	0/0	200 U/session × 1	10/2	28.57/33.33	-	-	-
16	5y3m	5y1m	24/3	BAC. 1#tid, SEL. 1#bid	24/3	0/0	-	-	-	STN-DBS (33ys/3y)	0/0	100/100
17	＞10y	3y10m	-	TIA. and DTBZ were ineffective	-	-	-	-	-	-	-	-

Improvement (%) was calculated as [(baseline score−post-treatment score)/baseline score]×100%； BTX dose is presented as units per session×number of sessions. Abbreviations: "-" indicates that the individual did not undergo the corresponding treatment; BTX, Botulinum Toxin; NA, not available; STN-DBS, Subthalamic nucleus deep brain stimulation; GPi-DBS, globus pallidus internus deep brain stimulation; ys, years old, y, years; m, months. AMA, amantadine; CZP, Clonazepam; DTBZ, Deutetrabenazine; BAC, Baclofen; L/B, Levodopa/Benserazide; THP, Trihexyphenidyl; TPM, topiramate; ZPD., Zolpidem; SEL., Selegiline; ARI., Aripiprazole; PGB., Pregabalin; ZNS., Zonisamide; OXC., Oxcarbazepine; L/C, Levodopa/Carbidopa; PPX, Pramipexole; FLX, Fluoxetine; CoQ10, Coenzyme Q10; TIA, Tiapride; GBP, gabapentin; ARO, arotinolol.
